# Potential for physician communication to build favorable medication beliefs among older adults with hypertension: A cross-sectional survey

**DOI:** 10.1371/journal.pone.0210169

**Published:** 2019-01-07

**Authors:** Song Hee Hong

**Affiliations:** 1 Social and Administrative Pharmacy, College of Pharmacy, Seoul National University, Seoul, Korea; 2 Research Institute of Pharmaceutical Sciences, Seoul National University, Seoul, Korea; Universite de Bretagne Occidentale, FRANCE

## Abstract

Older adults suffering from hypertension form firm medication beliefs through lifetime medication management, which significantly affect their medication adherence and treatment outcomes. Understanding whether the patient-physician communication has the potential to change medication beliefs will help design an effective communication strategy to foster favorable medication beliefs. This study aims to determine whether the patient-physician communication is associated with medication beliefs among older adults with hypertension and controls socio-demographics and clinical characteristics. Further, it examines how the association varies with two different types of medication beliefs (medication overuse and harm) for each domain of communication (informative and interpersonal). A self-administered cross-sectional survey was conducted for members of seven senior centers in a metropolitan area of the United States between August and December of 2013. A total of 211 senior members suffering from hypertension completed the questionnaire, which included the Primary Care Assessment Survey (PCAS) and the Beliefs about Medicines Questionnaire (BMQ). The former had two domains of patient-physician communication—informative and interpersonal—while the latter measured medication harm and overuse beliefs. Interpersonal patient-physician communication significantly explained the medication overuse beliefs (β = -0.28, p < 0.05), whereas neither interpersonal nor informative communication significantly explained the medication harm beliefs. Females (*β* = 1.29, *p* < 0.01) and participants with higher education (*β* = 2.66, *p* = 0.02) more strongly believed that medications are overprescribed. However, participants with low income more strongly believed that medications are harmful. Patient-physician communication, if it touches upon interpersonal aspects, has the potential to change medication overuse beliefs among older adults with hypertension. Identification of the significant factors which affect medication beliefs, will inform the design of a patient-centric communication program that fosters favorable medication beliefs among geriatric hypertensive patients.

## Introduction

Medication beliefs are known to affect medication adherence [1–4], an essential behavioral change required for successful treatment outcomes [5–7]. Therefore, physicians should think about ways to improve medication beliefs, as recommended by the good medical practice guidelines of the General Medical Council (GMC) in the United Kingdom [8]. It especially applies for chronic conditions such as hypertension, whose treatment requires long-term medication therapy.

A study reports that hypertensive patients have a different level of willingness to adhere to their respective physician’s medication recommendation [9]. It is highly likely that their medication beliefs affect their willingness to adhere. In fact, many studies report that medication beliefs significantly affect medication adherence [10–13]. Therefore, there is a critical need to identify the factors which are associated with medication beliefs. Identifying those factors can inform the design of effective strategies to improve medication beliefs for the purpose of better medication adherence.

Given that two-thirds of patients having hypertension are aged 60 years or older [14], it is important to examine the medication beliefs among older adults. Among them, medication belief exerts a stronger effect on medication adherence than among their younger counterparts [1, 3, 15]. This is likely because older patients hold firmer medication beliefs than their younger counterparts. Throughout their life, older adults have frequent encounters with medication use and, thus, will have formed firm medication beliefs. Especially while suffering from hypertension, they can become inflicted with many health complications and, thus, can be exposed to polypharmacy [16]. Older adults, then, are likely to believe that medication is overused. On the other hand, poor drug metabolism among older patients [17] makes the polypharmacy more harmful in terms of increasing the risk of adverse drug reactions [18–20]. Furthermore, older adults may have experienced more serious adverse drug reactions as medications used in the past contained more safety problems than the ones used nowadays. Thus, older adults with hypertension tend to believe that medication is overused and harmful.

With the advent of the paradigm of patient-centeredness in healthcare, patient-physician communication has gained attention owing to its potential of empowering patients to engage in behavior changes for better treatment adherence and outcomes [21]. The successful management of chronic diseases such as hypertension requires active patient engagement for better treatment adherence. Active patient engagement occurs when patient-physician communication helps the patient to form positive beliefs about treatment options. However, few studies have examined the relationship between physician-patient communication and patients’ medication beliefs among older adults with hypertension. This study aimed to determine whether patient-physician communication is associated with the medication belief as well as with patient characteristics among older patients with hypertension. It further aimed to compare the association between two types of medication beliefs (medication harm versus overuse belief) for each subdomains of patient-physician communication (PCAS-Communication versus PCAS-Interpersonal Treatment).

## Materials and methods

### Study design and data collection

A cross-sectional self-administered survey, consisting of two parts, was conducted between August 1, 2014 and December 31, 2014, using a convenience sample of elder adults aged 60 years or older, from seven senior centers situated in a metropolitan area of a southeastern state of the United States. All participants were asked to complete the first part of the survey, which included the Beliefs about Medicines Questionnaire-General (BMQ-General) and the Primary Care Assessment Survey (PCAS). The seniors who were taking any prescription drugs for high blood pressure were asked to complete the second part of the survey.

Before the survey was conducted, every senior center’s director was contacted in order to find an optimal date when many members of a given senior center would visit it and be able to participate in the survey. After contacting them, a letter was sent to each center’s director seeking for collaboration. Once the survey date was scheduled through collaboration, the center director was asked to broadcast the survey to its members at least a week before the date of the survey. On the survey date, two research assistants visited the center and conducted the survey in a reserved room. The potential study participants were informed that their participation was voluntary and that they could stop participating in the survey whenever they wanted to. For each participant who completed the survey, a grocery gift card of US $20 was given as a token of appreciation. The University of Tennessee Health Science Center Institutional Review Board (IRB) reviewed this survey and the informed consent via fast track. The IRB approved the study after having us remove the signature section at the bottom of the information sheet. The signature section was determined unnecessary because the survey was done anonymously. Requiring the study participants to sign would have made them fear that their identity could traced via the signature.

### Survey instruments

#### Patient-physician communication

Patient-physician communication was assessed using two subscales of the PCAS: Communication and Interpersonal Treatment [22]. The PCAS was devised to operationalize the definition of primary care outlined by the Institute of Medicine (IOM). The Communication scale looks at a physician’s communication regarding the patient’s medical history and symptom resolution, while the Interpersonal Treatment scale measures the patient-physician interaction with respect to aspects such as friendliness, care, and concern. More specifically, the Communication Scale asks patients to rate their doctors on six aspects of primary care: (1) the thoroughness of the questions being asked about their symptoms, (2) attention to what they have to say, (3) explanations provided about their medical conditions and treatment, (4) instructions about symptoms to report and when to seek help, (6) advice and help in making decisions about their own care, and (6) finally whether they leave their Physicians’ offices with unanswered questions. [22, 23]. The Interpersonal Treatment scale addresses another five aspects of primary care; (1) the amount of time spent with them, (2) patience with their questions, (3) friendliness and warmth received, (4) care and concern, and (5) finally the level of showed respect to them. Each item in the scales is rated on the Likert scale: Very poor = 1, Poor = 2, Fair = 3, Good = 4, Very good = 5, Excellent = 6; or Always = 1, Almost always = 2, A lot of the time = 3, Some of the time = 4, Almost never = 5, Never = 6). Each summated score is then normalized into a scale of 0–100. For this manuscript, the respective scales were named as informative and interpersonal communication.

#### Patient medication beliefs

The individual beliefs of the participants toward medication were measured using the Beliefs about Medicines Questionnaire-General (BMQ-General)[24]. The BMQ-general comprises two subscales (BMQ-harm and BMQ-overuse) (Table 1), each having 4 items in a 5-point Likert scale, with 1 being “strongly disagree” and 5 being “strongly agree.” The BMQ-harm measures the extent to which patients believe that medications are inherently harmful, while the BMQ-overuse assesses the excessive extent to which physicians place their trust in medications and, hence, overprescribe them. The BMQ-harm and BMQ-overuse scales have scores in the range of 4 to 20, where the higher scores indicate more negative perceptions.

**Table 1:** Contents of beliefs about medicines questionnaire.

Categories	Questions
BMQ-Harm	Stop taking medicines every now and again Addiction Property Harm over benefit Poison
BMQ-Overuse	Doctors’ prescribing Doctors trusting on medicine Time with patients or medicines Natural remedies or medicines

Abbreviation: *BMQ*, Beliefs about Medicines Questionnaire.

#### Patient characteristics

The socio-demographic characteristics that were measured included the patients’ age, gender, education, race, household income, cohabitants, marital status, and comorbidity. Their age and household income were measured via open-ended questions. The incomes were then grouped into four levels according to the standards of the state of Tennessee [25]. To measure the burden of comorbid diseases, the Charlson Comorbidity Index (CCI) was employed.

### Statistical analysis

Descriptive statistics about the socio-demographic and clinical characteristics of the hypertensive participants were summarized by using the mean and the standard deviation for continuous variables and the frequency and the percentage for categorical variables. Multiple linear regression analyses were performed to examine the predictability of patient-physician communication on the patients’ medication beliefs, which control patient characteristics. Then, the regression coefficient (β) and the standardized beta coefficient (Β) were presented. P values of less than 0.05 were considered statistically significant. All statistical analyses were conducted using SAS 9.4 (SAS Institute, Inc., Cary, NC, USA).

## Results and discussions

### General characteristics of the study sample

Of the 300 participants who were recruited for the survey [26], the number of the participants who completed both the first and the second section of the survey was 211 (Table 2). Among them, 79% were over 65 years old, with more than two-thirds being female (77.3%). Almost all (96.7%) of them had an educational background of attending high school or a higher institution. There were about 10% more non-Hispanic whites (52.6%) than non-Hispanic blacks (42.2%). Almost half (51.4%) had an income lower than US $29,531. On an average, the study participants had suffered from hypertension for a period of 11.6 years (*SD* = 8.5) with one-third (38.9%) having a comorbidity of 0 in CCI, and 31.8% CCI having a comorbidity of 2.

**Table 2:** Description of study participants.

Participant Characteristics	No. of Participants ^a^ (n = 211)	Percent (%)
**Age**		
Not elderly (≤ 64)	44	21.1
Young old (65–75)	101	48.3
Medium or Oldest old (≥ 76)	64	30.6
**Sex**		
Male	48	22.7
Female	163	77.3
**Education**		
≤ Middle School	7	3.3
High school or graduate	88	42.1
Some college	65	31.1
≥ college graduate	49	23.4
**Race**		
Non-Hispanic White	111	52.6
Non-Hispanic Black	89	42.2
Others^b^	11	5.2
**Income**		
Low (< $29,531)	94	51.4
Lower middle ($29,531–$44,297)	34	18.6
Middle ($44,294–$88,594)	46	25.1
High (≥ $88,594)	9	4.9
**Living status**		
Alone	89	42.6
With Spouse	77	36.8
Others^c^	43	20.6
**Marital status**		
Married	79	37.4
Divorced/Separated	48	22.7
Widowed	69	32.7
Never married	15	7.1
**Comorbidity class**		
0	82	38.9
1	62	29.4
2	67	31.8
**Disease duration (hypertension) Mean (SD)**	211	11.6 (8.5)

Notes:

^*a*^For some variables, the total is less than 211 due to missing values.

^*b*^Others include Asian, Indian, Pacific islanders, or Alaskan natives.

^*c*^Others include living with other family members (daughter or son, sister or brother, mother, grandson or pets) or companion.

SD: standard deviation.

### Description of communication and medication belief scales

The participants of the study reported that their communication with the physician was satisfactory, i.e., the mean PCAS score was 76.5 (SD = 15.6) out of 100 for the informative communication and 76.4 (SD = 15.2) for the interpersonal communication. In terms of medication beliefs, the study participants had a stronger belief regarding medication overuse (mean = 12.4; SD = 2.9) than on medication harm (mean = 8.9; SD = 2.9). In other words, the study participants more strongly believed the notion that medications are overprescribed than the view that medications are harmful (Table 3).

**Table 3:** Patient-reported outcomes.

Self-reported outcome	Mean (SD)	Range
Patient-physician communication (n = 211)		
Informative domain	76.5 (15.6)	25–100
Interpersonal domain	76.4 (15.2)	36.7–100
BMQ-General (n = 211)		
BMQ-Overuse	12.4 (2.9)	4–20
BMQ-Harm	8.9 (2.9)	4–18

Abbreviations: *BMQ*: Beliefs about Medicines Questionnaire; *SD*: standard deviation.

### Patient-physician communication on patient medication beliefs

**Fig 1:**
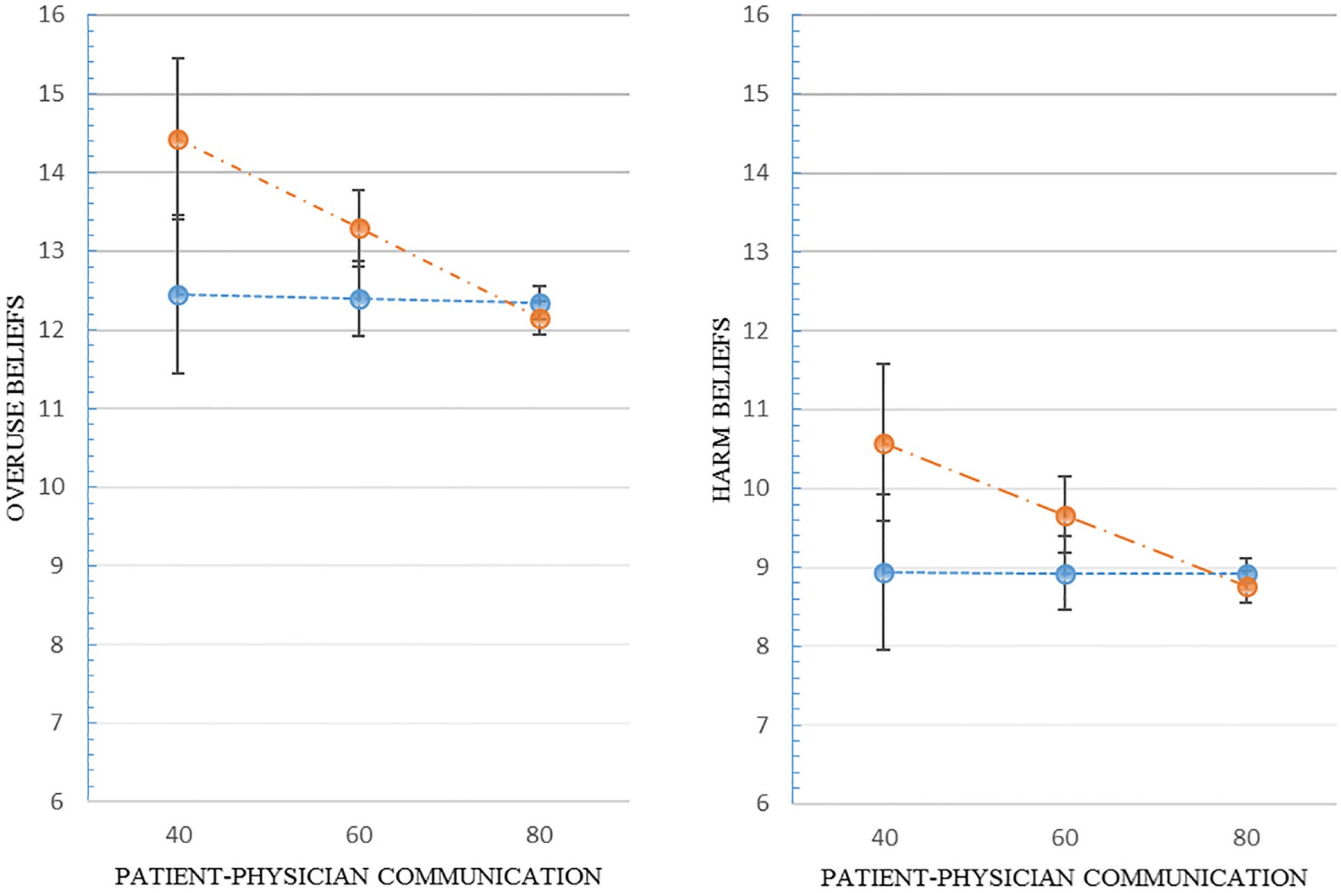
The effect of patient-physician communication on patients’ beliefs of medication overuse and harm. Orange Dots: Interpersonal Communication; Blue Dots: Informative Communication.

Regarding the associations of physician communication types with medication beliefs, interpersonal communication had a significant association with the belief of medication overuse, i.e., an increase in interpersonal communication was significantly associated with a decrease in medication overuse belief (β = -.05; p = 0.05) (Table 4). However, interpersonal communication was not significantly associated with the medication harm belief (β = -.04; p = 0.09). On the other hand, the informative communication was not associated either with the medication overuse belief nor with the medication harm belief. A graphical depiction of the predicted values of medication overuse and harm beliefs over a range of scores of each physician communication summarizes the way in which each domain of physician communication is associated with the respective medication beliefs (Fig 1).

**Table 4:** Predictability of patient-physician communication on medication beliefs.

	BMQ-overuse			BMQ-harm		
	β^a^	STD β^b^	P value	β	STD β	P value
(Intercept)	14.97	0	< 0.0001	13.31	0	< 0.0001
**Age**						
Non-elderly (≤ 64)	Reference					
Young old (65–75)	-0.24	-0.04	0.66	0.10	0.02	0.86
Medium or Oldest old (≥ 76)	-0.32	-0.05	0.61	0.12	0.02	0.85
**Gender**						
Male	Reference					
Female	**1.29**	**0.19**	0.01**	0.74	0.11	0.12
**Education**						
≤ Middle School	Reference					
High school or graduate	1.90	0.32	0.09	0.12	0.02	0.91
Some college	**2.68**	**0.43**	0.02*	0.37	0.06	0.75
≥ College graduate	**2.66**	**0.39**	0.02*	-0.00	-0.00	1.00
**Race**						
Non-Hispanic White	Reference					
Non-Hispanic Black	0.44	0.07	0.32	0.44	0.08	0.31
Others	-0.55	-0.04	0.54	0.42	0.03	0.63
**Income**						
Low	Reference					
Lower middle	-0.16	-0.02	0.78	-0.01	-0.00	0.98
Middle	0.46	0.07	0.41	**-1.12**	**-0.16**	0.04*
High	-0.76	-0.05	0.45	**-2.39**	**-0.17**	0.02**
**Living status**						
Alone	Reference					
With spouse	-1.49	-0.25	0.32	-0.96	-0.16	0.52
Others	0.82	-0.11	0.13	-0.66	-0.09	0.22
**Marital status**						
Married	Reference					
Divorced/Separated	-0.44	-0.06	0.77	-1.17	-0.17	0.42
Widowed	-0.74	-0.12	0.62	-1.57	-0.26	0.28
Never married	-2.43	-0.21	0.14	-1.57	-0.14	0.34
**Comorbidity class**						
0	Reference					
1	-0.86	-0.14	0.08	0.41	0.07	0.39
2	-0.69	-0.11	0.15	-0.39	-0.06	0.41
**Patient—physician communication**						
Informative domain	-0.00	-0.03	0.84	-0.00	-0.00	0.98
Interpersonal domain	**-0.05**	**-0.28**	0.05*	-0.04	-0.25	0.09
**Mean period of hypertension**	0.02	0.06	0.35	-0.02	-0.07	0.28
R^2^	0.27			.25		

Notes:

^a^β means regression coefficient.

^b^STD β means standardized beta coefficient.

*: *p* < .05,

**: *p* < .01

Abbreviations: Low (< $29,531), Lower middle ($29,531–$44,297), Middle ($44,294–$88,594), High (> $88,594).

### Patient characteristics’ effect on patient medication beliefs

The patients’ socio-demographic factors were significantly associated with both subdomains of medication beliefs: BMQ-Overuse and BMQ-harm beliefs (Table 4). Gender had a significant association with BMQ-Overuse, i.e., females were more likely to believe that doctors overprescribe medications (β = 1.29; p = 0.01). Education too had a positive association with BMQ-Overuse. The participants with some amount of college education (β = 2.68; p = 0.02) as well as those with educational backgrounds of at least a college degree (β = 2.66; p = 0.02) had a stronger medication overuse belief as compared to those having an education less than or equal to the attendance of middle school. Regarding the BMQ-harm belief, participants in the middle-income bracket (β = -1.12; p = 0.04) as well as those in the high-income bracket (β = -2.39; p = 0.02) had a stronger belief that medication is harmful, as compared to those in the low-income bracket.

### Discussions

This study found that among the two types of medication beliefs, only the medication overuse belief was significantly explained by patient-physician communication that specifically pertained to the interpersonal domain. Physician communication influences medication beliefs is a widely held opinion. In fact, it is widely reported that physician communication is key to improving treatment adherence and outcomes [27]. What our study adds to the literature is that it is the interpersonal domain not the informational one that significantly explains the medication overuse belief. Given as many as 9.8 prescriptions each visit to a family doctor produces, it is natural that older adults hold the medication overuse belief [28]. This medication overuse belief, however, if not alleviated, could potentially hinder good medication adherence [29]. Our study finding informs of the importance of interpersonal communication to alleviate the medication overuse belief. The interpersonal communication intends to build trust with patients while the informative one concerns the verbal delivery of information related to specific tasks [30]. The patient who trusts their physician would not believe that medication is overused. To the contrary, the patient who understands the medication would not necessarily give up the medication overuse belief.

Given that an office physician’s visit reportedly lasts less than 15 minutes [31, 32], patients are likely to leave their physicians having many of their questions left unanswered [33]. This then leads to patients distrusting their physicians [34], which amplifies the belief of medication overuse. In this practice environment of churning out patient visits, physicians who want to foster favorable medication overuse beliefs should communicate interpersonally with their patients and exhibit emotional support, for example, by displaying friendliness, warmth, and concern towards them. Meanwhile, the medication harm belief was not significantly explained by any domain of patient-physician communication. More specifically, the association between the medication harm belief and the interpersonal communication (p = 0.9) did not reach the statistical significance of p = .05. The medication harm belief is likely formed through the patient experience of medication treatment. The medication harm belief thus is inherent to medication while being less prone to physician communication. Future studies need to examine whether interpersonal communication, has the potential to alleviate the medication harm belief when the communication specifically focuses on medication.

Regarding other predictors of medication beliefs, the lowest-income bracket had the strongest beliefs pertaining to medication harm. People with lower income are less likely to have access to high-quality healthcare goods and services [35]. As a result, they are more likely to develop these harm beliefs [36, 37]. Furthermore, they are more likely to participate in clinical trials irrespective of safety concerns for better financial rewards and, thus, are more prone to experiencing the harmful effects of medications [38–41].

As for education, it was significantly associated with medication overuse belief but not with medication harm belief. People with higher education reportedly have more physician visits than people with lower educational background and, thus, they are more likely believe that physicians overprescribe medications. However, this requires an explanation of why education had no significant association with the belief pertaining to medication harm. Perhaps, education impacts the harm belief in two opposite ways. On one hand, education makes people more aware of the presence of harmful effects while on the other hand, it helps them avoid using harmful medications. Those two influences offset each other, leading to no significant association between education and medication harm belief.

As for gender difference, females were found to have a significantly stronger medication overuse belief than males. This gender difference may have originated from higher healthcare utilization among females. Females are known to make more visits to physicians and hospitals than males [42]. Thus, they receive more medications [43, 44] as compared to males [44]. They also tend to provide more specific and clear explanations about their symptoms, which results in them having more prescriptions for medication.

### Limitations

There are several potential limitations in our study. The generalizability can be limited, as the study participants were recruited from a metropolitan region in a southeastern state of the United States. For example, the study participants were mostly females (77.3%). However, it was not far removed from a typical senior center [45]. A previous study of several senior centers in a metropolitan region in the United States reports that 80.4% of its participants were female [46], which can be explained due to their longer life expectancy, earlier retirement, and more willingness to move around [47].

This study did not use the BMQ-Specific instrument for medication beliefs. The BMQ-Specific concerns patients’ beliefs about specific medications that are prescribed to each patient. The BMQ-General, on the other hand, concerns patients’ beliefs about all medications which are being used in practice. BMQ-General is correlated with BMQ-Specific [24, 48]. However, BMQ-General is for people who do not have a common condition or a specific treatment, as in our study [49].

## Conclusions

Patient-physician communication, specifically the interpersonal one, significantly explained the medication overuse beliefs among older patients having hypertension, while neither interpersonal nor informative communication could explain the medication harm beliefs. Regarding other factors which affected medication beliefs, education and gender were found to significantly explain medication overuse beliefs. However, lower income was significantly associated with stronger medication harm beliefs. This study concludes that understanding the potential of patient-physician communication to affect medication beliefs would inform the design of future studies for effective patient-tailored communication strategies for older hypertensive patients.

### Implications

Patients’ medication beliefs can affect their perceptions of and attitudes towards their medications. Thus, the finding that patient-physician communication has the potential to change patients’ medication beliefs could have important implications with respect to improving their drug-taking behaviors. Given that hypertensive patients regularly visit their physicians, patient-physician communication, when tailored to the needs of individual patients, helps them form favorable medication beliefs and show better medication-taking behaviors. Recognition of different patient characteristics that may affect this potential of patient-physician communication will inform the design of tailored communication strategies that are aimed at improving medication treatment outcomes.

## Supporting information

S1 File Analysis data file. (PRN)
